# Formulation and *In-Vitro* Evaluation of Ocular Ciprofloxacin-Containing Minitablets Prepared with Different Combinations of Carbopol 974P and Various Cellulose Derivatives

**Published:** 2010

**Authors:** Seyed Alireza Mortazavi, Zahra Jaffariazar, Elnaz Damercheli

**Affiliations:** a *School of Pharmacy, Shaheed Beheshti University of Medical Sciences, Tehran, Iran.*; b * School of Pharmacy, Islamic Azad University, Tehran, Iran.*

**Keywords:** Ocular minitablets, Ciprofloxacin, Sustained drug release, Cellulose derivatives, Carbopol 974P, Kinetic models

## Abstract

The aim of this study was preparation and evaluation of ciprofloxacin-containing minitablets for ocular use, in an attempt to obtain prolonged and controlled drug release to the anterior eye segment.

Following initial studies on ciprofloxacin powder, it was formulated into ocular minitablets. For this purpose, ciprofloxacin along with various amounts of different sustained release cellulose derivatives (HPMC, Na CMC, HEC and EC), Carbopol 974P, solubilizer and lubricant were directly compressed into minitablets, using concave 3 mm diameter punches.

All the prepared formulations were evaluated in terms of physicochemical tests, including uniformity of weight, friability, crushing strength, water uptake and in-vitro drug release studies.

It was found that the type and amount of cellulose derivatives used, can influence the rate of drug release.

The finally selected formulation (B_3_) contained ethyl cellulose, Carbopol 974P, mannitol, sodium stearyl fumarate and ciprofloxacin, which showed more than 80% drug release over a period of 5h, and complied well in all the physicochemical tests conducted.

Based on kinetic studies, formulation B_3_ was found to best fit the zero order equation. However, the Higuchi and Hixson-Crowell models also showed a good fit. Hence, overall formulation B_3_ was chosen as the best formulation.

## Introduction

The use of novel ocular drug delivery systems has found an important role in the treatment and management of various ophthalmic diseases, over the past two decades. In this respect, numerous factors can affect ophthalmic drug delivery, including rapid drainage, blinking reflex, lacrymation and low corneal permeability. In addition, intrinsic properties of the drug and the type of dosage form used, can influence the process of drug delivery (1, 2).

Most conventional ophthalmic dosage forms are simplistic. It is usual that water- soluble drugs are delivered through topical administration in an aqueous solution, and water insoluble drugs are administered topically, as an ointment or aqueous suspension.

The major deficiencies of these conventional dosage forms include poor ocular bioavailability, pulse-drug entry after topical administration, systemic exposure due to nasolacrimal duct drainage, lack of effective dosage forms for drug delivery to the posterior segment of ocular tissue, high instillation frequency, poor patient compliance, toxic side effects and cellular damage at the ocular surface.

Optimization of ocular therapy can be performed by developing innovative drug delivery systems, for the purpose of precorneal drug retention, followed by an increased drug bioavailability (3-5).

In previous studies, ocular bioadhesive minitablets have been developed and optimized, showing sustained drug release properties (6-12).

Among the novel ocular drug delivery systems, which have been proposed and studied in recent years, are minitablets. In a study, the influence of compression force on the formation of minitablets illustrated that increasing the compression force leads to a decline in friability and swelling capacity, and an increase in the crushing strength (7).

In another study, the effect of different sterilization methods (gamma-irradiation and dry heat) on minitablets showed that the method used can affect the properties of minitablets. It was stated that gamma-irradiated minitablets containing ciprofloxacin could be considered as a promising formulation to treat bacterial keratitis and conjunctivitis (8).

In a separate study, which investigated the effect of roller compaction setting on the preparation of ocular minitablets, the results showed that the roller speed and the compaction force have the largest influence on the characteristics of the resulting granules. Regarding the tablet strength, friability and dissolution profile, a low compaction force and a high roller speed were shown to be preferable to prepare minitablets (9).

Characterization of minitablets prepared with different Carbopol-starch components, illustrated that higher viscosity values were obtained for sterilized co-spray dried powder mixtures containing an amount of Carbopol 974P equal or above 15 % w/w, compared to the physical blends. The amount of Carbopol 974P in the co-spray dried powder mixtures, as well as the use of gamma-irradiation method for sterilization, had no influence on the crushing strength and friability of the minitablets (11). 

The present study investigated the use of matrix-type systems containing hydrophilic polymers, as a new strategy to obtain the ciprofloxacin minitablets and evaluation of their properties. Ciprofloxacin, belonging to the 4-quinolones family of antibacterials, was used as the model drug in this study. The hydrophilic polymers investigated in this study, were the popular cellulose derivatives as well as Carbopol 974P. These polymers have been used in various formulations, but not in ocular minitablets (13-15).

The minitablets prepared in this study were characterized in terms of uniformity of weight, friability, water uptake, crushing strength, in-vitro drug release and kinetics of drug release.

## Experimental

**Materials**

Hydroxypropylmethyl cellulose (HPMC, 4000 cps), sodium carboxymethyl cellulose (NaCMC, 1300-1500 cps), ethyl cellulose (EC, 100 cps), and hydroxyethyl cellulose (HEC, 300 cps) were all purchased from Acros Co. (Geel, Belgium). Carbopol 974P was obtained from Noveon (New York, USA). Sodium stearyl fumarate (NaSF) was from JRS Pharma (Madrid, Spain). Ciprofloxacin hydrochloride hydrate was gifted by Temad Co. (Tehran, Iran). Mannitol was supplied by the Merck Co. (Darmstadt, Germany). The isotonic phosphate buffer solution (pH 7.4) was prepared using 4.30 g sodium dihydrogen phosphate dihydrate and 16.25 g disodium hydrogen phosphate dehydrate, both obtained from the Merck Co. (Darmstadt, Germany).

*Preparation of minitablets*

Table 1 presents the composition of minitablets prepared in this study. The powder mixtures consisted of ciprofloxacin (3% w/w), cellulose derivatives (90-95% w/w), Carbopol 974P (3-5 % w/w), and NaSF (1 % w/w), which were homogeneously mixed, individually, using pestle and a mortar. Following thorough mixing, the powder mixtures were separately compressed into 3 mm convex minitablets, weighing 7 mg, using an eccentric tablet press (Korsch-EKO model, Berlin, Germany) at the highest possible force. The production method was based on direct compression.

**Table 1:** Composition of the ocular ciprofloxacin containing minitablets prepared in this study

**Ingredients**	**Group A formulatios (%)**				**Group B formulations (%)**		
	**A**_1_	**A**_2_	**A**_3_	**A**_4_	**B**_1_	**B**_2_	**B**_3_
HPMC	91	-	-	-	-	-	-
NaCMC	-	91	-	-	-	93	-
HEC	-	-	91	-	46.5	-	-
EC	-	-	-	91	46.5	-	72
Ciprofloxacin	3	3	3	3	3	3	3
Carbopo l974P	5	5	5	5	3	3	4
NaSF	1	1	1	1	1	1	1
Mannitol	-	-	-	-	-	-	20

*Characterization of the minitablet formulations prepared*

The prepared minitablets were evaluated in terms of various physicochemical tests including the uniformity of weight, crushing strength, friability, water uptake and in-vitro drug release, as explained below. 

*(I) Uniformity of weight*

The uniformity of weight of the prepared minitablets was determined by accurately weighing 10 tablets individually, using an electrical balance with an accuracy of 0.001 g (Mettler, Germany), and then calculating the average weight and standard deviation (16, 17). 

*(II) Crushing strength*

The behavior of 10 ocular minitablets from every prepared batch was analysed under an applied force, using a tablet hardness tester (Model TBH28, Erweka, Germany).

*(III) Friability test*

The method used for the determination of friability of ocular matrix minitablets prepared in this study, was based on previously described methods (7, 11, 12). The friability of the matrix minitablets (F_m_) was determined by initially weighing 10 tablets together. Next, the tablets were placed alongside 100 glass beads (average diameter of 4 mm) in a Pharma test friabilator (Model S 48-3 cm, Iran), set at a speed of 25 rpm, and faced falling shocks for 10 min. After 10 min, the glass beads were removed, and tablets were re-weighed in order to determine the percentage of friability, based on the following equation (7-9, 11) : 

F_m_ (%) =100 × (*P *− *P*´) /*P                     *(equation 1)

Where *P *= initial weight of 10 minitablets and *P*´ = final weight of 10 minitablets.

*(IV) Water uptake*

The amount of water uptake or in other word the extend of hydration of the prepared minitablets was determined at room temperature, gravimetrically.

Accurately weighed minitablets (M_d_) were individually placed on the upper side of a glass-filter, which was itself placed on the lower side of a reservior filled with pH 7.4 isotonic buffer phosphate solution. The minitablets were completly sank within the solution. The weight of the swollen (hydrated) minitablets (M_w_) was determined at set time intervals, at room temperature, until the weight of tablet remained unchanged or even reduced. In order to calculate the percentage of water uptake (*W *%), equation 2 was used (6, 7, 9) :

$\mathrm{W\%}=100\times \frac{M_{w}-M_{d}}{M_{d}}$                     (equation 2)

M_d_ = initial weight of the dry minitablet, and M_w_ = final weight of the dry mini tablet.

*(V) In-vitro drug release*

The release of ciprofloxacin from various series of formulated minitablets was examined using glass vials in an oscillating water bath.

Each minitablet was accurately weighed, and then transferred into a glass vial containing 1mL pH 7.4 isotonic phosphate buffer. In order to avoid water evaporation, the vials were covered with rubber caps. They were then placed in an oscillating (25 rpm) water bath at 32 ± 1°C. Throughout the experiment, 300 μL aliquots were withdrawn at 20, 40, 60, 90, 120, 180, 240, 300 and 1440 min time intervals, and subsequently replaced by an equal volume of pH 7.4 isotonic phosphate buffer (7-9, 11, 12).

In order to determine the amount of ciprofloxacin released from the studied minitablets, a calibration curve of ciprofloxacin was constructed in pH 7.4 isotonic phosphate buffer. The absorbance values were measured using a UV spectrophotometer (UV-VIS 1201, Shimadzu, Japan) at 270 nm. The obtained calibration curve was found to be linear (y = 0.0783x- 0.002, R^2^ = 0.9998).

The percentage of drug released at each time interval was expressed as a fraction of the total amount of drug present within the minitablets.

The drug release profile obtained from the selected formulation was evaluated kinetically (Table 2), using the zero order, first order, Higuchi, Korsmeyer-Peppas and Hixson-Crowell models (19-21). Excel 2000 (Microsoft, Redsmond, USA) software was used for calculation of the release rate constants (k_x_), with the aid of solver tool.

**Table 2:** The mathematical models used to investigate the kinetics of drug release in this study.

**Mathematical model**	**Formula**
Zero order	Q_t_ = k_0_ t
First order	In Q_t_ = In Q_0 _+ k_1 _t
Higuchi	$Q_{t}=K_{H}\sqrt[{2}]{t}$
Hixson- Crowell	$\sqrt[{3}]{Q_{0}}-\sqrt[{3}]{Q_{1}}=K_{\mathrm{HC}}$t
Korsmeyer- Peppas	Q_t_/Q_∞_ = K_KP_ t^n^

Q_t_ = total amount of drug dissolved in time t; Q_0_= initial amount of drug within the tablet; Q_t_/Q_∞ _= fraction of drug released in time t; n = defines the mechanism of release profile based on the Fick’s law.

*(VI) Statistical analysis*

Statistical evaluation of the different properties of the formulated minitablets was performed, using the one-way analysis of variance (ANOVA), along with the Tucky post test. For this purpose SPSS version 15.0 software was used. A statistical significance was defined at p < 0.05. 

## Results and Discussion

Different characteristics of the minitablet formulations (groups A and B) prepared were investigated and will be presented and discussed as follows.

*Physical characterization of group A ciprofloxacin minitablets*

As mentioned in Table 1, the formulations prepared in group A were made of a fixed amount of Carbopol 974P (5 % w/w), along with 91 % w/w of various cellulose derivatives. In addition, they all contained 3 % w/w ciprofloxacin and 1 % w/w NaSF (as lubricant).

The physical properties of the minitablets prepared in group A, have been summarized in Table 3.

**Table 3:** Physical characteristics of ocular ciprofloxacin minitablets prepared in group A (mean value ± SD).

**Formulation**	**Weight (mg) ** **n=20**	**Crushing strength (N) ** **n=10**	**Friability (%)** **n=3**	**Water uptake (%)** **n=3**
A_1_	7.00 ± 0.10	7.30 ± 0.14	0.96 ± 0.83	1253.50 ± 76.60
A_2_	6.35 ± 0.49	11.50 ± 0.21	1.40 ± 0.15	2683.50 ± 41.72
A_3_	6.30 ± 0.47	4 .90 ± 0.10	1.53 ± 2.17	1427.00 ± 127.67
A_4_	6.35 ± 0.49	10.50 ± 0.27	4.01 ± 0.88	2757.10 ± 177.14

The weight variation (acceptable range of ±10 % ) and crushing strength (acceptable range of 1-18 N) of all the formulations prepared in group A are within the acceptable limits, based on the existing standards and published data (7-9, 11, 16, 17). The mean weight of formulation A_1_ is the highest and shows a significant difference with the other formulations (p < 0.05). The reason for this finding could be the larger particle size and density of HPMC than the other polymers (22). 

Generally speaking, addition of Carbopol has managed to produce an adhesive nature in all the formulations prepared, consequently helps to provide integrity and compactness within the resulting minitablets. 

In contrary, the friability values of all group A minitablet formulations (except A_1_) were above the acceptable limit of 1%.

Based on the results obtained, formulations A_2_, A_3_ and A_4_ showed higher friabilities than formulation A_1_, however not statistically significant (p > 0.05).

When considering the extent of water uptake and the swelling behavior of the minitablet formulations prepared in group A, formation of a hydrated gel layer around the surface of the minitablets was observed. The examined minitablets showed a considerable increase in their dimensions upon contact with the aqueous medium.

The greatest amount of water uptake was observed in formulation A_4_, followed by formulations A_2_, A_3_ and A_1_. Statistical analysis illustrates a significant difference among the extent of water uptake between all formulations, except for A_1_ & A_3_, and A_2_ & A_4_. Despite the hydrophobic nature of EC polymer, it has the ability to form water uptaking channels within the matrix network. Hence, it helps with the greater rate of absorbed water by the minitablet matrix. 

The mechanism of water uptake by the other formulations is different.

In formulation A_2_ which contains NaCMC, as a hydrophilic and anionic polymer capable of creating a higher osmotic pressure, water uptake is more than formulations A_1_ and A_3_ which contain non-ionic cellulose derivatives. Furthermore, B_3_ formulation shows a higher amount of water uptake than A_1_. It seems that the greater hydrophilic nature of HEC than HPMC is the reason for this difference (22).

Finally, when considering the dissolution profiles of group A minitablet formulations, 80 % (or greater) drug release within 5 h was defined as the acceptable limit. 

**Figure 1:**
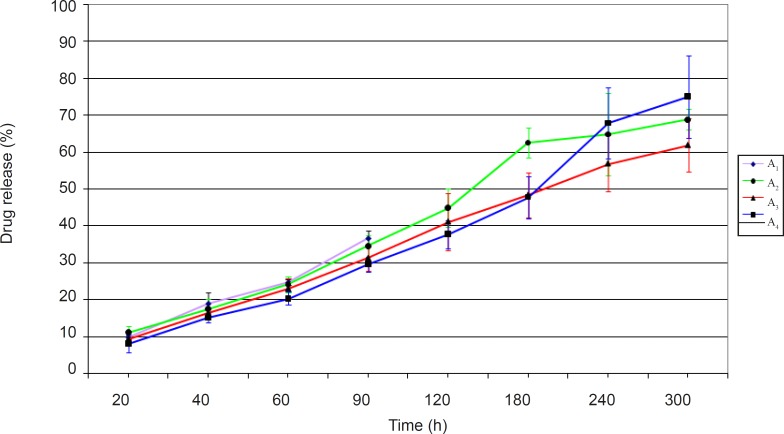
Release profiles of ciprofloxacin from group A minitablets in pH 7.4 isotonic phosphate buffer at 32 ± 1°C (n = 3, mean ± SD).

The ciprofloxacin release profiles obtained from minitablets studied in group A have been shown in Figure 1.

Figure 1 shows that the dissolution profiles are different for the formulations studied. The noticeable point in release profiles obtained is the correlation between the amount of drug released and the extent of water uptake by the test formulation. The greater the amount of water uptake, the higher would be the amount of drug release. In fact the highest amount of drug release in group A formulations belonged to formulation A_4_. It seems that hydrophilic channels that have been formed by the EC polymer as well as the anionic Carbopol, would allow a greater degree of water entrance into the matrix network, consequently helping to diffuse out ciprofloxacin to a higher extent from the minitablet formulation. Formulation A_1_ was found unsuitable, since complete disintegration of minitablet occurred after 2 h. 

In addition, formulation A_2_ and A_3_ did not release a sufficient amount of drug within 5 h. Between these two formulations, formulation A_2_ which contained the hydrophilic and anionic water absorbent NaCMC, showed a greater amount of drug release than formulation A_3_ which contained the non-ionic HEC.

Overall, based on the drug release studies conducted, none of the formulations prepared in group A ciprofloxacin minitablets were found to be acceptable. 

*Physical characterization of group B ciprofloxacin minitablets*

Minitablet formulations prepared in group B were made of 72-93 % w/w cellulose derivatives, 34 % w/w Carpobol 974P, 1 % w/w NaSF, 20 % w/w mannitol (in formulation B3) and 3 % w/w ciprofloxacin.

The physical properties of the minitablets prepared in group B are summarized in Table 4. 

**Table 4:** Physical characteristics of ciprofloxacin minitablets prepared in group B (mean ± SD).

**Formulation**	**Weight (mg)** **n=20**	**Crushing strength (N) n=10**	**Friability (%)** **n=3**	**Water uptake (%)** **n=3**
B_1_	6.75 ± 0.78	8.20 ± 0.16	1.71 ± 1.51	2049.85 ± 310.80
B_2_	7.10 ± 0.96	15.90 ± 0.29	1.11 ± 1.00	2618.13 ± 192.48
B_3_	6.90 ± 0.55	15.90 ± 0.35	0.53 ± 0.42	2156.49 ± 92.15

In this group only the weight variation of formulation B_3_ was found to be within the acceptable limit of ± 10 %. This is presumably due to the presence of mannitol in this formulation, enhancing the flow of the powder mix into the die acavity.

On the other hand, in formulations B_1 _and B_2_ the presence of cellulose derivatives, with poor flowability, could result in non-uniform filling of the die cavity and hence a greater weight variation. 

The crushing strength of all the group B formulations were found to be suitable. This means that decreasing the amount of Carbopol 974P within the minitablet, does not influence the crushing strength.

Furthermore, the good compactibility of mannitol present within formulation B_3_ can result in the increased crushing strength of this formulation, compared with the corresponding formulation A_4_ with no mannitol present.

The addition of mannitol to formulation B_3_ leads to a decrease in the friability of this formulation, compared with formulations B_1_ and B_2_, which did not contain mannitol. However, the differences observed were not statistically significant (p > 0.05). Overall, in terms of friability, only formulation B_3_ with a value smaller than 1% was found to be within the acceptable limit of friability, but not formulations B_1_ and B_2_. 

The amount of water uptake by group B formulations were in the ascending order of B_2_ >B_3 _>B_1_ (i.e. formulation B_2_ had the greatest amount of water uptake). Statistical analysis of the results showed a significant difference between the amount of water uptake by formulation B_2_, compared with the other two formulations (p < 0.05).

It seems that the presence of the anionic cellulose derivative, NaCMC, would enhance the amount of water entering the minitablet matrix to a far greater extent than the non-ionic HEC and EC. Moreover, addition of the hydrophilic water absorbing mannitol alongside the hydrophobic EC in formulation B_3_, would increase the amount of water uptake by this formulation slightly more than formulation B_1_ with no added mannitol. 

**Figure 2:**
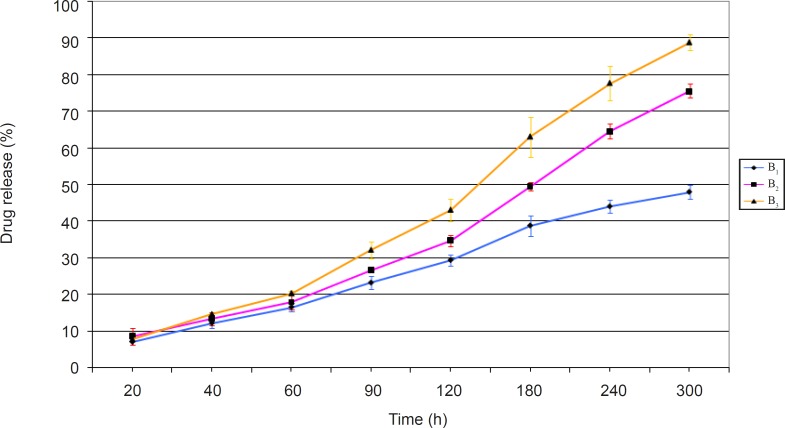
Release profiles of ciprofloxacin from group B minitablet formulations in pH 7.4 isotonic phosphate buffer at 32 ± 1°C (n = 3, mean ± S.D).

The release profiles of ciprofloxacin obtained from evaluating the prepared minitablet formulations of group B have been presented in Figure 2.

The results obtained (Figure 2) showed that formulation B_3_ had the highest amount of drug release, and 88 % of its drug content was released after 5 h. This was the greatest among all formulations of groups A and B. In other words, the inclusion of mannitol in formulation B_3_ would help to create fine pores within the matrix network. Hence, it improves the rate of water intake and consequently drug release from this formulation. On the contrary, formulation B_1_ showed the lowest amount of drug release among group B formulations. This means that the addition of the hydrophilic non-ionic polymer, HEC, alongside the hydrophobic polymer, EC, can not produce the same amount of drug release observed with formulation B_3_ which contained EC alongside mannitol. Although the release profile of formulation B_2_ was not acceptable, it showed a higher release than formulations B_1_ and A_2_ (the corresponding formulations in group A). NaCMC is an anionic polymer, capable of providing a greater osmotic pressure within the matrix network. Hence, its presence in formulation B_2_ would help to increase the amount of drug release, compared to formulation B_1_, which does not contain this polymer.

Statistical analysis of the release profiles obtained among group B formulations, also showed a significant difference between the results obtained (p < 0.05).

Overall, based on the results obtained, formulation B_3_ seems to have all the desirable properties. Hence, it was selected as the best formulation among both groups of formulations prepared and underwent kinetic studies. 

*Kinetic studies on the release profile of selected formulation B*_3_

The release rate constants (k) and correlation coefficients (R) calculated by fitting various mathematical models (mentioned in Table 2) into the drug release profile of formulation B_3_ have been summarized in Table 5.

**Table 5:** Release rate constants and correlation coefficients obtained after fitting various mathematical models into the release profile of formulation B_3_.

**Mathematical model**	**K**	**R** ^2^	**n**
Zero order	17.9460	0.9882	-
First order	0.4783	0.8630	-
Higuchi	53.6680	0.9882	-
Korsmeyer- Peppas	21.6421	0.9972	0.9227
Hixson- Crowell	0.4907	0.9551	-

As could be seen in Table 5, formulation B_3_ seems to fit both the zero order and Higuchi model of drug release. However, considering the “n” value (index) obtained for formulation B_3_ using the Korsmeyer-Peppas mathematical model, which was found to be equal to 0.9227, it appears that a zero order model of drug release can better define the mechanism of drug release from this matrix-type minitablet (i.e. n > 0.89 corresponds to a zero-order kinetic of drug release). Nevertheless, since the profile of drug release also fits the Higuchi model to the same extent as the zero-order mathematical model, it is feasible that formulation B_3_ has a complex kinetic of drug release, following both the stated models at different stages of drug release. This means that the minitablet matrix can swell and later on starts to erode, consequently releasing its drug content.

In conclusion, it could be said that the use of hydrophilic polymers and in particular a combination of Carbopol 974P and EC, as found in this study, alongside the pore-forming mannitol can be used as a successful matrix for the preparation of ocular extended release minitablets.
